# Measurement uncertainty analysis of single-use flow sensors

**DOI:** 10.3389/fbioe.2025.1455336

**Published:** 2025-03-26

**Authors:** Niclas Ludolph, Julian Haller, Andreas Prediger

**Affiliations:** ^1^ PD PAT and Flow Kits, Sartorius Stedim Biotech GmbH, Goettingen, Germany; ^2^ Metrology, Sartorius Lab Instruments GmbH & Co. KG, Goettingen, Germany

**Keywords:** single-use sensors, flow sensor, measurement uncertainty, bio pharma, bio process

## Abstract

The increased application of single-use (SU) equipment and sensor technology in biopharma and bioprocesses has a significant impact on development and production. User are faced with the question how sensor uncertainties of SU devices can be handled and estimated to ensure process reliability. The classical methods by means of calibration and resulting uncertainty determination are only transferable to SU sensors to a limited extent. Sensor elements are often delivered pre-assembled and without the possibility of in-process calibrations. Moreover, manufacturers’ specifications do not use strictly defined terms but constructions such as “1-sigma accuracy”, which makes it even more difficult for the end customer to determine sensor uncertainties. The purpose of this article is to demonstrate the determination of measurement uncertainty using a 1/2-inch BioPAT®Flow SU sensor as an example to accomplish a comparison with the accuracy specification given in the sensor data sheet. A linear regression could be determined as the upper limit of the combined measurement uncertainty: $u\left(Q\right)=0.0114\;Q+0.182\;L/\min$ .

## 1 Introduction

Single-use (SU) equipment is widely used in modern biopharmaceutical production facilities for up- and downstream applications and replaces multi-use (MU) equipment from individual measuring components to entire SU systems. Such equipment is designed for a single production run and enables shorter development and production cycles. SU equipment can be pre-sterilized (e.g., via ionizing radiation), which renders laborious time- and energy-intensive sterilization procedures at production sites obsolete. Thus, biopharmaceutical production using SU equipment can operate with lower environmental impacts and at better cost-efficiency (Pietrzykowski et al., 2013; Saxena, 2018).

Detailed process monitoring and control is only possible with adequate sensor concepts that are adapted to the requirements of SU production processes. Reliable measurement results are required to make production processes reproducible (Bissig et al., 2020). Sensor accuracy is crucial, as increasing the number of sensors enhances data reliability but increases costs (Liu et al., 2023; Shi et al., 2022). This is accompanied by a growing demand for process qualification and, thus, for the determination of measurement uncertainty.

Several SU sensor concepts exist for disposable bioreactors (Busse et al., 2017; Eibl and Eibl, 2019; Olsen et al., 2017) and downstream systems (Eibl and Eibl, 2019; Olsen et al., 2017; Eibl and Eibl, 2019). Metrological challenges arise depending on the different interface solutions of the sensors to the SU system. While some SU sensors can be calibrated immediately before usage, this is not possible for others due to technical or economic considerations. On the one hand, the costs of proper calibration might be higher than the costs of the SU sensor itself, and on the other hand, proper calibration would contaminate the sensor and/or the process. Moreover, SU sensors often consist of a disposable port or flow cell acting as an interface to the SU system and a reusable transmitter. In such cases, the final sensor is formed by joining the two parts before usage, which needs to be considered when determining the measurement uncertainty “in use.”

There is currently no common industry standard for characterizing the performance of SU sensors. Different manufacturer specifications, which are generally not directly comparable, are used. According to the International Vocabulary of Metrology [“VIM” (JCGM 200, 2012)], “the concept ‘measurement accuracy’ is not a quantity and is not given a numerical quantity value.” Nevertheless, manufacturers in the bioprocess industry often specify by characterizing their measurement systems instead of uncertainty. Although it is still common to stipulate numerical values for the “accuracy” of a sensor, this work will rather use terminology according to common normative documents like the VIM or the Guide to the Expression of Uncertainty in Measurement (JCGM 100, 2008), particularly the terms “measurement uncertainty,” “combined uncertainty,” “standard uncertainty,” and “expanded uncertainty” as defined in these documents (JCGM 200, 2012; JCGM 100, 2008).

To assess the accuracy or uncertainty of a single-use sensor that includes both single-use and multi-use components, it is essential to evaluate not only the accuracy of the multi-use component (such as a transmitter) but also to incorporate the single-use component and the interaction between these components.

## 2 Background

### 2.1 Sensor technology

**FIGURE 1:**
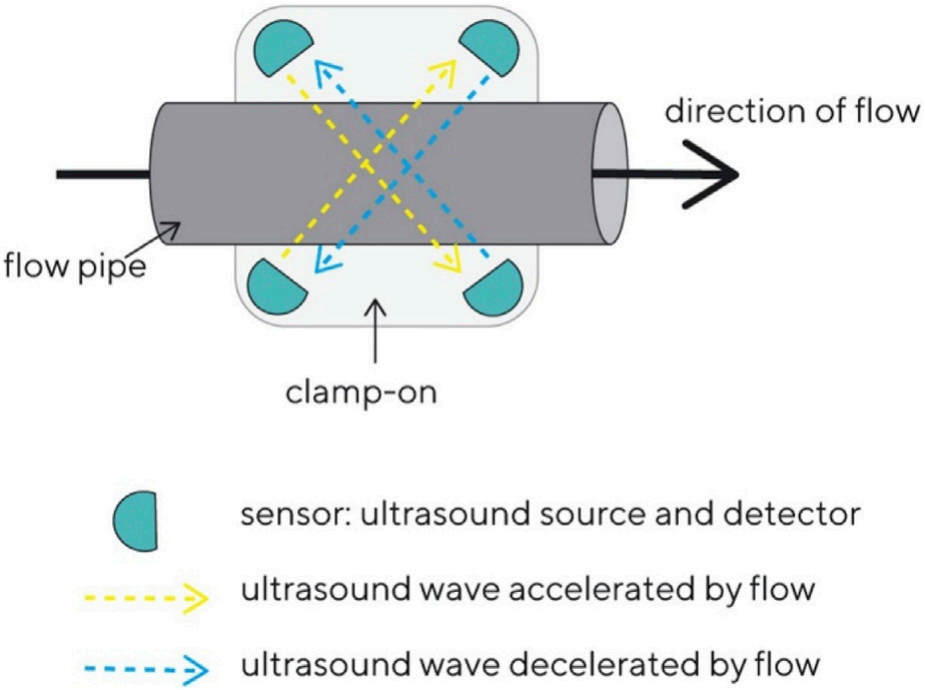
Schematic principle of the function of the BioPAT®Flow sensor.

The BioPAT^®^ Flow sensors (Sartorius product) use ultrasonic transit time technology to determine the flow rate. Figure 1 depicts the measurement principle.

Ultrasound signals travel back and forth between transducer pairs that are diagonally mounted in the clamp-on sensor. The ultrasound signals travel through the liquid and are either accelerated or decelerated by the flow stream. This leads to a transit time difference between the two signals that is proportional to the average flow velocity, from which the flow rate can be calculated using the inner diameter of the flow pipe.

**FIGURE 2:**
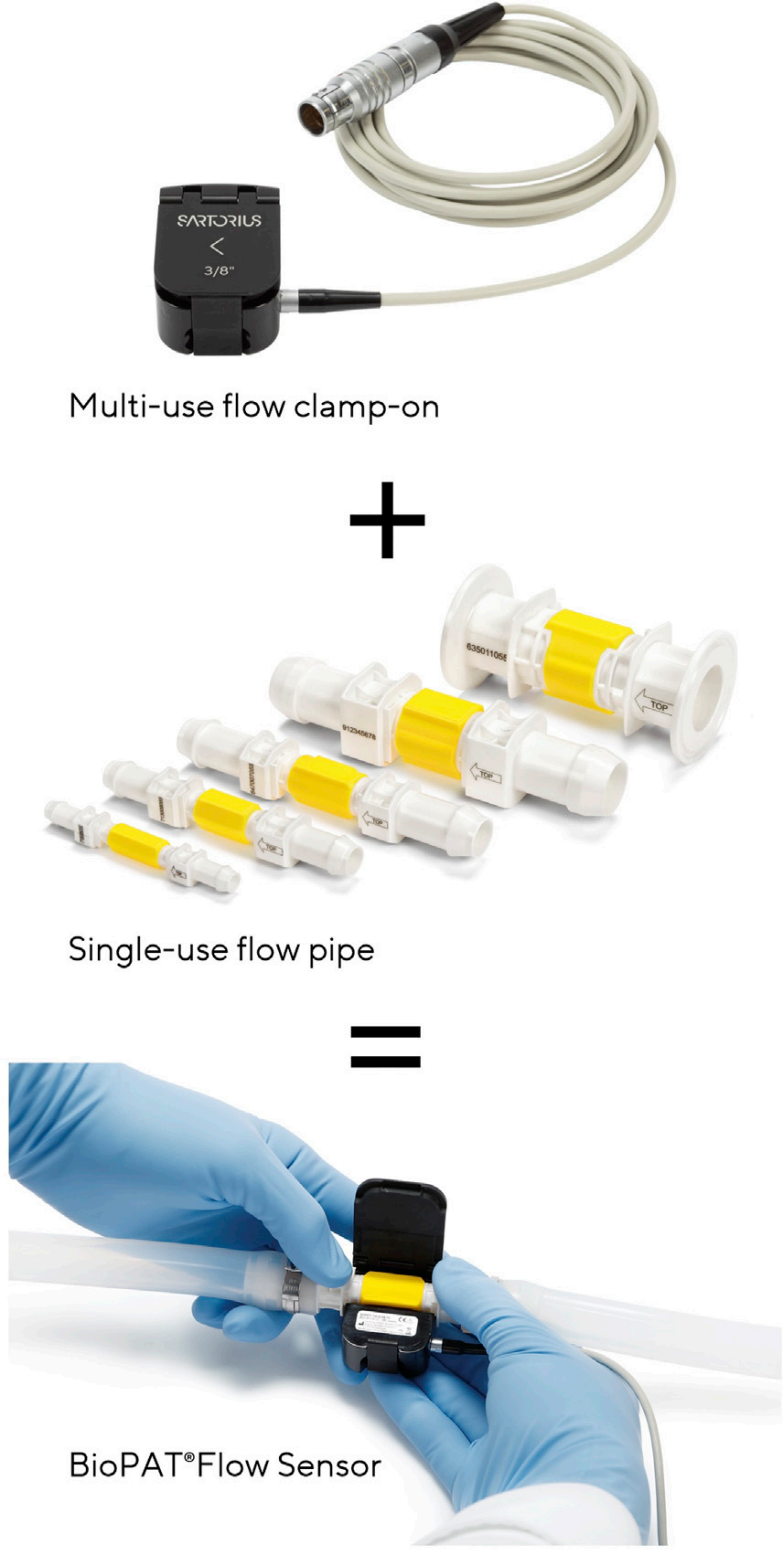
Multi-use flow clamp-on, SU flow pipe, BioPAT®Flow Sensor.

The flow pipes are integrated into SU tube assemblies. The clamp-on transmitter is fixed around the pipe, allowing for the measurement to be carried out (Figure 2).

## 3 Methods

### 3.1 Master meter method

In addition to the gravimetric method for calibration, there is also the master meter method. Compared to the gravimetric method, the master meter method has the advantage of enabling the measurement of pressure levels and the possible tempering of the entire assembly. The master meter method compares two different sensors for the same measured quantity and thus uses one as a test device for the other. The sensors are used in the same measurement setup in the same test. Thus, one sensor serves as a reference for the other to be calibrated (Chun et al., 2017; Hogendoorn et al., 2011; Pattasseril et al., 2013).

**FIGURE 3:**
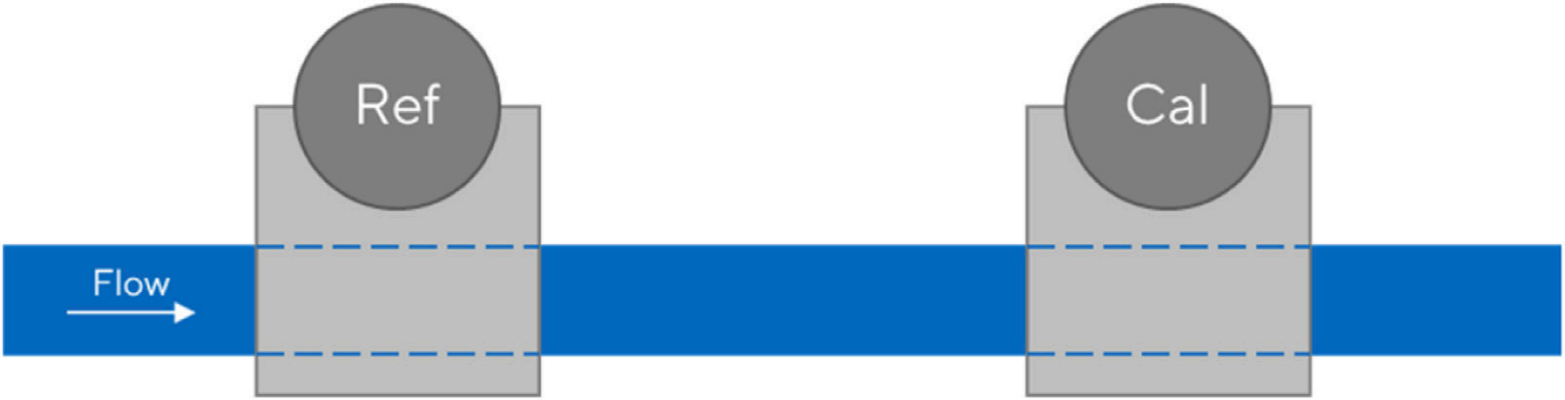
Schematic representation of the master meter method for flow sensors.

Numerous calibration laboratories worldwide use this method for volumetric flow rate measurements because it is less complicated than the gravimetric method, and the measurement setup is easier to implement. Traceability to the corresponding SI unit can be established via the reference sensor, if appropriately calibrated. This method is illustrated in Figure 3.

### 3.2 Measurement setup

The following setup was chosen for testing the BioPAT^®^ Flow sensors. The test liquid was fed from a temperature-controllable reservoir via a pump (PSG Quatro Flow 5050 S) to three consecutive BioPAT^®^ Flow sensors.

An ABB HygienicMaster300^®^ served as the reference flow sensor downstream of the SU sensors. Before the liquid was returned to the reservoir, which was adjusted via a diaphragm valve (GEMÜ 618), the temperature and pressure were measured. The measurement setup was designed with piping and a reference sensor matching the inner diameter of 1/2 inch from the BioPAT^®^ Flow sensors.

### 3.3 Uncertainty model

The “reference sensor calibration” contribution is considered via use of the master meter method. For a detailed mathematical uncertainty analysis for this aspect of flowmeters, we recommend the article “How Accurate are Ultrasonic Flowmeters in Practical Conditions: Beyond the Calibration” (JCGM 200, 2012).

This article places a larger emphasis on the contributions of SU sensors and process parameter-related aspects.

In addition to repeatability and reproducibility, which are contributions for all kinds of measurement equipment (and defined in Sections 3.2, 3.3), the SU-related uncertainty contributions are here separated into the four following aspects (marked with quotation marks). “Due to different transmitters” relates to the effect of different transmitters combined with one flow pipe. “Due to different SU batches” encompasses the effects of different flow pipes in combination with the same electronic periphery. The coupling of a transmitter with a flow pipe also affects the uncertainty budget and is titled here as “Contribution of coupling.” “Instrumental drift” sums up the impact of the storage of the SU parts on the uncertainty budget.

The combined uncertainty is derived from the single uncertainty contributions following the GUM (JCGM 100, 2008). As determination of the intrinsic influence of the single contributions on the measurement principle is hardly possible, a statistical approach is chosen here instead, where the single contributions are considered as additive errors ∂_Qi_ to the measured value Q_meas_:

$$Q=Q_{\text{meas}}+\sum_{i=1}^{n}\partial Q_{i}.$$ (1)

Furthermore, for the uncertainty analysis chosen here, no correlation between the single uncertainty contributions is considered because of the measurement data discussed later. Basically, the formula is simplified and serves as an upper estimate of the uncertainty budget. In summary, we obtain Equation 2 for the combined uncertainty by applying Equation 10 of (JCGM 100, 2008) to the above Equation 1:

$$u\left(Q\right)=\sqrt{\begin{array}{c} {\left(u_{\text{cal}}\left(Q\right)\right)}^{2}+{\left(u_{\text{repeat}}\left(Q\right)\right)}^{2}+{\left(u_{\text{repro},\text{SU}}\left(Q\right)\right)}^{2}\\ +{\left(u_{\text{repro},\text{Trans}}\left(Q\right)\right)}^{2}+{\left(u_{\text{repro},\text{Coup}}\left(Q\right)\right)}^{2}{+\left(u_{t_{x}}\left(Q\right)\right).}^{2}\\ +{\left(u_{T}\left(Q\right)\right)}^{2}+{\left(u_{M}\left(Q\right)\right)}^{2}+{\left(u_{q}\left(Q\right)\right)}^{2}+{\left(u_{p}\left(Q\right)\right)}^{2} \end{array}}$$ (2)

The uncertainty contributions are listed in detail in the Table 1 below and in the following sections.

**TABLE 1:** Classification of uncertainty contributions taken into account for the considerations.

Source of uncertainty	Description
Reference sensor calibration	
Calibration $u_{\text{cal}}\left(Q\right)$	Uncertainty due to reference flow sensor calibration
Sensor-related aspects	
Repeatability $u_{\text{repeat}}\left(Q\right)$	Random variation due to the operator, the measurement setup, place, system, …
Reproducibility 1. $u_{\text{repro},\text{SU}}\left(Q\right)$ 2. $u_{\text{repro},\text{Trans}}\left(Q\right)$ 3. $u_{\text{repro},\text{Coup}}\left(Q\right)$	1. Variation due to different SU components 2. Variation due to different transmitters 3. Variation due to coupling
Component Aging $u_{t_{x}}\left(Q\right)$	Uncertainty due to aging
Process parameter-related aspects	
Temperature $u_{T}\left(Q\right)$	Uncertainty due to temperature
Media $u_{M}\left(Q\right)$	Uncertainty due to media
Flow rate $u_{q}\left(Q\right)$	Uncertainty due to flow rate
Pressure $u_{p}\left(Q\right)$	Uncertainty due to pressure

## 4 Materials and methods

### 4.1 Reference sensor calibration

The calibration uncertainty $u_{\text{cal}}\left(Q\right)$ of the master meter method is sufficiently described by Chun et al. (JCGM 200, 2012). The method and setup for calibrating the SU sensors investigated in this article are described in Sections 3.2, 3.3. The respective uncertainty of the reference sensor should generally be taken from its respective calibration certificate.

### 4.2 Sensor-related aspects

The repeatability of a measurement is defined as the “measurement precision under a set of repeatability conditions of measurement” (JCGM 200, 2012).

Due to the partial automation of the setup, many possible sources of error that could be introduced by the operator are eliminated. However, the environmental and measurement conditions may change during the measurement, which may introduce an uncertain amount of repeatability $u_{\text{repeat}}\left(Q\right)$ to the measurement result. Due to this definition of repeatability uncertainty, these contributions are included over other uncertainty factors.

The reproducibility of a measurement is defined as the “condition of measurement, out of a set of conditions that includes different locations, operators, measuring systems, and replicate measurements on the same or similar objects” (JCGM 200, 2012). The measurement made for the contribution caused by the SU unit is illustrated in Figure 8, where a transmitter was coupled with different SU components. The SU component is, therefore, the variable. This measurement includes, for example, manufacturing-related variances. The contribution is hereinafter referred to as $u_{\text{repro},\text{SU}}\left(Q\right)$ .

**FIGURE 4:**
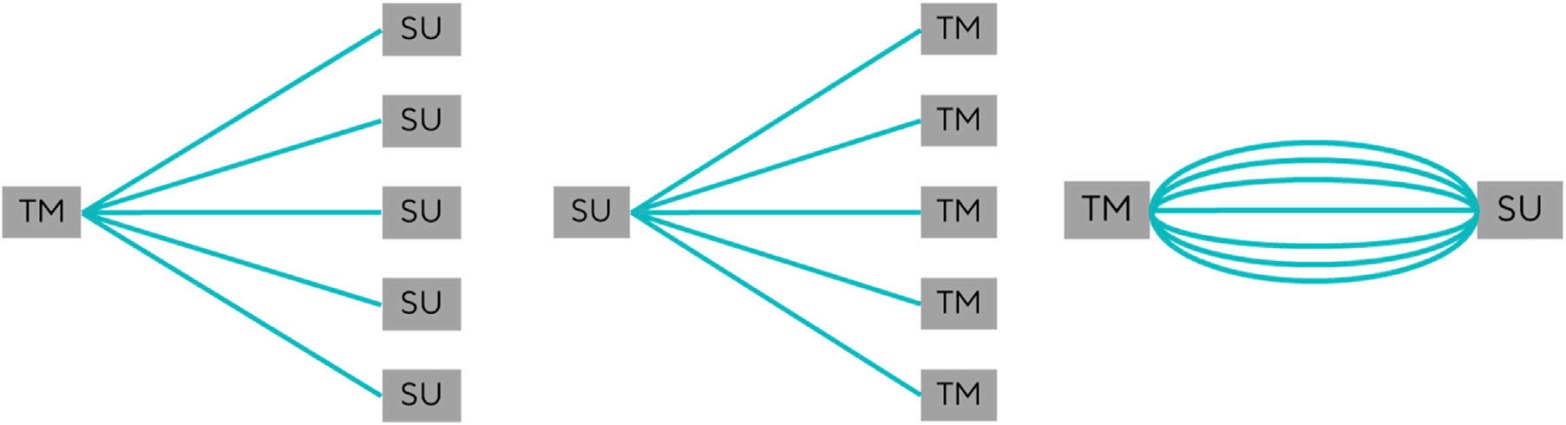
One transmitter subsequently coupled with multiple SU pipes, one SU pipe subsequently coupled with multiple transmitters, and one transmitter subsequently coupled multiple times with the same SU pipe.

Different transmitters can be used to determine the flow rate. To investigate the influence of transmitter variability, multiple transmitters of the same type were tested with the same SU flow pipe. This provides information about the reproducibility of the measurement and the uncertainty contribution caused by transmitters (Figure 4). The contribution is hereinafter referred to as $u_{\text{repro},\text{Trans}}\left(Q\right)$ .

Attaching the transmitter to the SU pipe also affects the measurement results. The uncertainty contribution caused by this coupling is illustrated in Figure 4. In these measurements, the same transmitter was repeatedly connected to the same SU pipe. The contribution is hereinafter referred to as $u_{repro,Coup}\left(Q\right)$ .

Immediately after production of a device, it usually complies with the specifications. However, storage and aging of the product (i.e., instrumental drift) can have an impact on the quality of the product and should be taken into consideration. For the product qualification of the BioPAT^®^ Flow, a shelf-life study was carried out, and the data from this study were considered within this article. The uncertainty contribution due to instrumental drift of the flow measurement is referred to as $u_{t_{x}}\left(Q\right)$ in the following work.

The process parameters of the system and their influence on it were considered to determine uncertainty. For the determination of Type A uncertainty, measurement series were carried out. The influence of different volumetric flow rates, temperatures, media, pressures, and instrumental drift effects of the SU components were examined.

The BioPAT^®^ Flow sensors have three standard calibration tables that must be chosen to fit the desired temperature range of the monitored process liquid. The standard calibration tables have a range of ±5°C around a defined calibration point. To investigate the uncertainty contribution of temperature differences of the process media, measurements were carried out at the center point and at the ends of a calibration table (i.e., at 17°C, 22°C, and 27°C). This uncertainty contribution is referred to as $u_{T}\left(Q\right)$ in the following.

Ultrasound propagates at different speeds and with different dispersion angles in different media. Gases can be dissolved in the liquid, which can lead to artifacts in the measurement (Tanaka et al., 2001). Ultrasonic flow sensors are best suited for media similar to water (Engel, 2013; Frøysa et al., 2015). Because only water and bovine serum albumin (BSA) were tested in the experiments, we point out the effects on the uncertainty contribution as $u_{\rho }\left(Q\right)$ throughout this article, but we only refer to the differences between these investigated media.

In this case, the volume flow rate under test additionally influences itself. Its uncertainty contribution $u_{q}\left(Q\right)$ is caused, among other things, by turbulence of the flowing medium (Frøysa et al., 2015). A detailed mathematical description would make the model very complex. Therefore, measurements were carried out at different flow rates to estimate this contribution instead, while the sensor and the flow pipe were not changed.

### 4.3 Pressure

Different pressure levels could affect the behavior of the flow pipes (e.g., via deformation of the pipe). A rigid material (polybutylenterephthalate, PBT) was chosen to produce the flow pipes to minimize the pressure influence. Nevertheless, the uncertainty contribution of the pressure to the flow measurement was investigated and is referred to as $u_{p}\left(Q\right)$ in the following.

Several other possible uncertainty contributions were considered and found to be negligible under the employed conditions, including the resolution of the sensor results (being on the order of 0.001 L/min).

## 5 Measurements and results

The measurements performed and their respective results are presented in the following sections. The contribution of each uncertainty source to the combined measurement uncertainty is calculated.

### 5.1 Reference sensor calibration

The “accuracy” statement in the calibration sheet of the reference sensor is provided by the calibration service provider using Equation 3:

0.2 % o.m.+0.02 % o.e.. (3)

Here, o. m. is the respective measuring point, and o. e. is the measuring range end point of the reference sensor (45 L/min). A qualified calibration certificate with respective uncertainty values would have been more meaningful, but due to the lack of such a certificate, the stated “accuracy” is taken here as a tolerance value, that is, of Type B.

As this contribution can be considered a Type B uncertainty with a rectangular probability distribution, it is multiplied by $\frac{1}{\sqrt{3}}$ . Equation 4 is used to obtain a standard uncertainty, as described in Section 4.3.7 of JCGM 100 (2008):

$$u_{\text{cal}}\left(Q\right)=\frac{\left(0.002\cdot Q+0.009\;L/\min\right)}{\sqrt{3}}$$ (4)

### 5.2 Sensor-related aspects

#### 5.2.1 Reproducibility

The following determined reproducibility uncertainty values of different SU components and different transmitters include effects of the reproducibility of coupling efficiency. Nevertheless, both contributions are fully considered without any correlation reduction to obtain a conservative estimation of the overall uncertainty.

Furthermore, the following determined reproducibility uncertainty contributions are assumed to be independent of the measured flow rate. This may, however, be different for sensor principles, where the strength of the measured signal is proportional to the final measurement and should be investigated separately in such a case.

#### 5.2.2 Reproducibility of different SU components

To determine the influence of different SU components (flow pipes), 15 different flow pipes from different batches were tested (all with the same transmitter) against a reference sensor. The measurements were performed at a flow rate of 10 L/min and a media temperature of 22°C.

**FIGURE 5:**
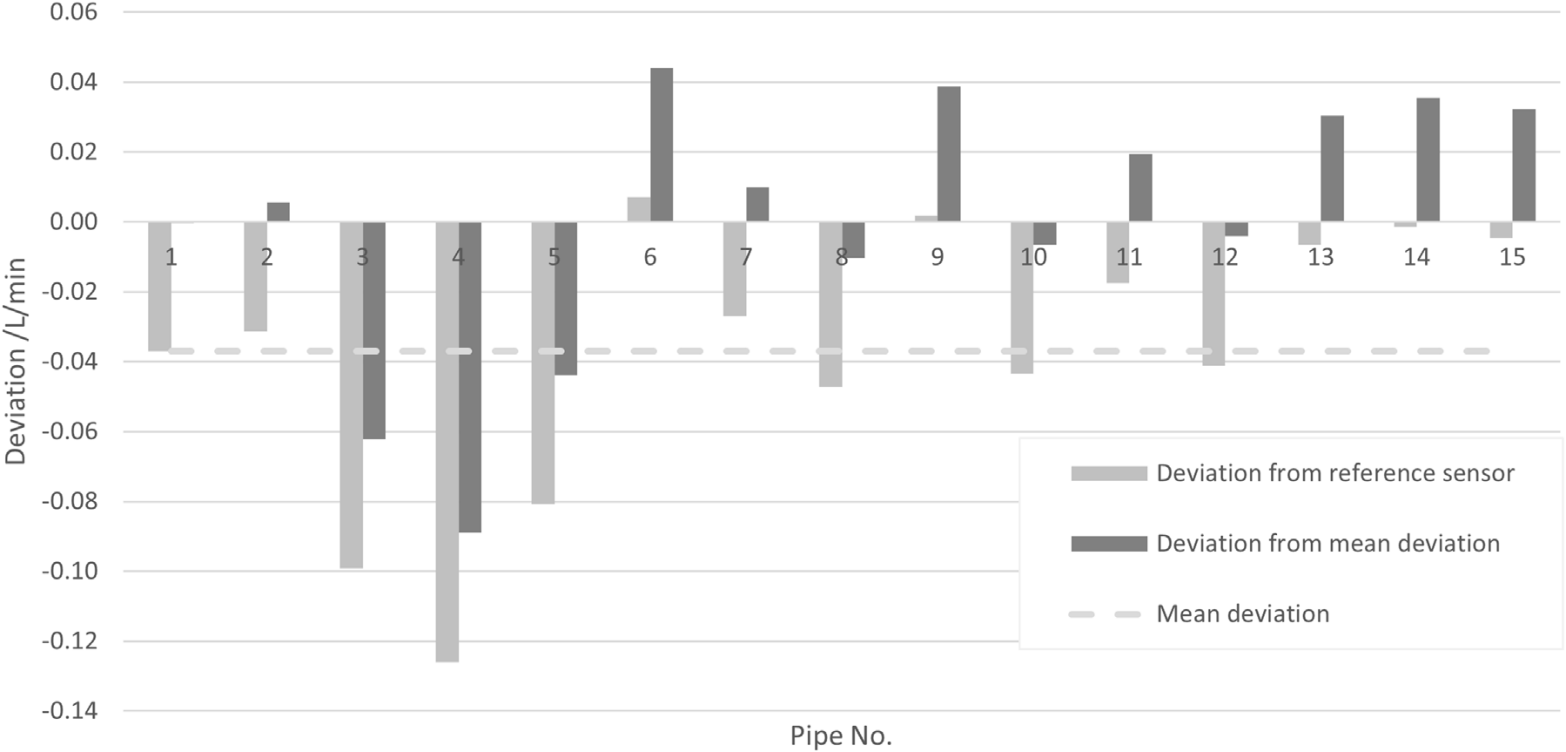
Deviations of the measurement result for different pipes with the same transmitter to the result of a reference sensor (light gray bars) and deviation to their mean (dark gray bars).

Figure 5 shows the mean deviation of the measurement with each of the 15 different pipes to the measurement with the reference sensor (light gray bars). In order to separate the deviations due to the different pipes from the systematic error of the employed transmitter, the deviations from the mean deviation (i.e., averaged over the 15 pipes) are also shown (dark gray bars). The uncertainty contribution reflecting the random variation of different pipes is estimated as the standard deviation of the 15 deviations, which yields $u_{\text{repro},\text{SU}}\left(Q\right)=$ 0.041 L/min for the presented data.

#### 5.2.3 Reproducibility of different transmitters

**FIGURE 6:**
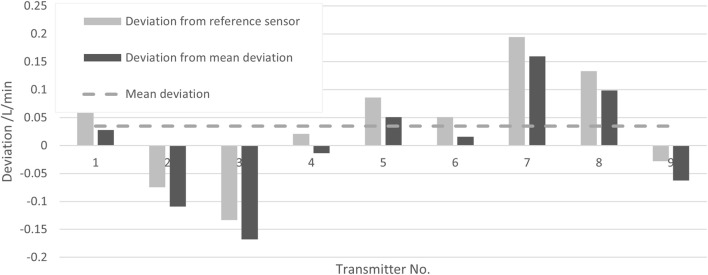
Deviations of the measurement result for different transmitters with the same pipe to the result of a reference sensor (light gray bars) and deviation to their mean (dark gray bars).

To determine the uncertainty contribution of the transmitters on the measurement, nine different transmitters were all coupled to the same flow pipe. The measurements were performed at a flow rate of 10 L/min and 22°C media temperature. In Figure 6, the deviations of the measurement results with the different transmitters (all with the same pipe) to the results obtained with a reference sensor are plotted as light gray bars, while the deviations from their mean—eliminating the systematic error—are plotted as dark gray bars.

The standard deviation of these values is taken as the uncertainty contribution reflecting the reproducibility due to different transmitters, yielding $u_{\text{repro},\text{Trans}}\left(Q\right)$ = 0.097 L/min for the presented data.

#### 5.2.4 Reproducibility of coupling

**FIGURE 7:**
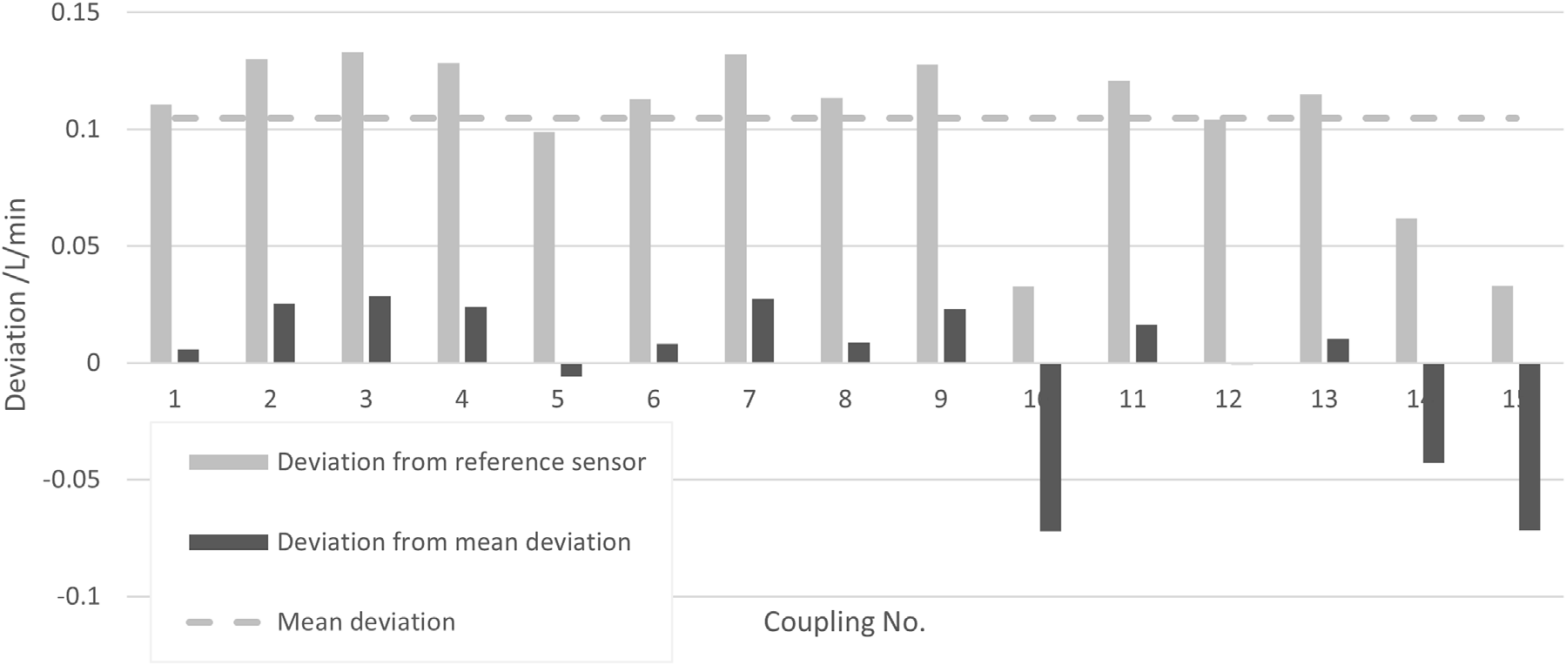
Deviations of the measurement result for several couplings of the same transmitter with the same pipe from the result of a reference sensor (light gray bars) and deviation from their mean (dark gray bars).

The repeated process of connecting a single transmitter to the same flow pipe was investigated. The measurements were performed at a flow rate of 10 L/min and a media temperature of 22°C. The coupling was performed 15 times. In Figure 7, the deviations of the measurement results of the re-coupled SU system from the results obtained with a reference sensor are shown as light gray bars, and the deviations of the successive couplings to their mean are shown as dark gray bars.

The standard deviation of these values is taken as the uncertainty contribution reflecting the reproducibility due to the coupling of a transmitter with a pipe, yielding $u_{\text{repro},\text{Coup}}\left(Q\right)$ = 0.033 L/min for the present data.

#### 5.2.5 Instrumental drift

With regards to the aging of sensor components, it was assessed that the impact of aging on the uncertainty contribution would be higher in the SU components than in the multi-use components (clamp-on transmitters). Thus, only the instrumental drift effect on the SU components is investigated below, while the instrumental drift effect on the clamp-on transmitters is neglected. The following aging levels of the SU flow pipes were investigated.

T0) without aging.
T1) 1 year accelerated aging.
T3) 3 years accelerated aging.
T6) 6 years accelerated aging.

**FIGURE 8:**
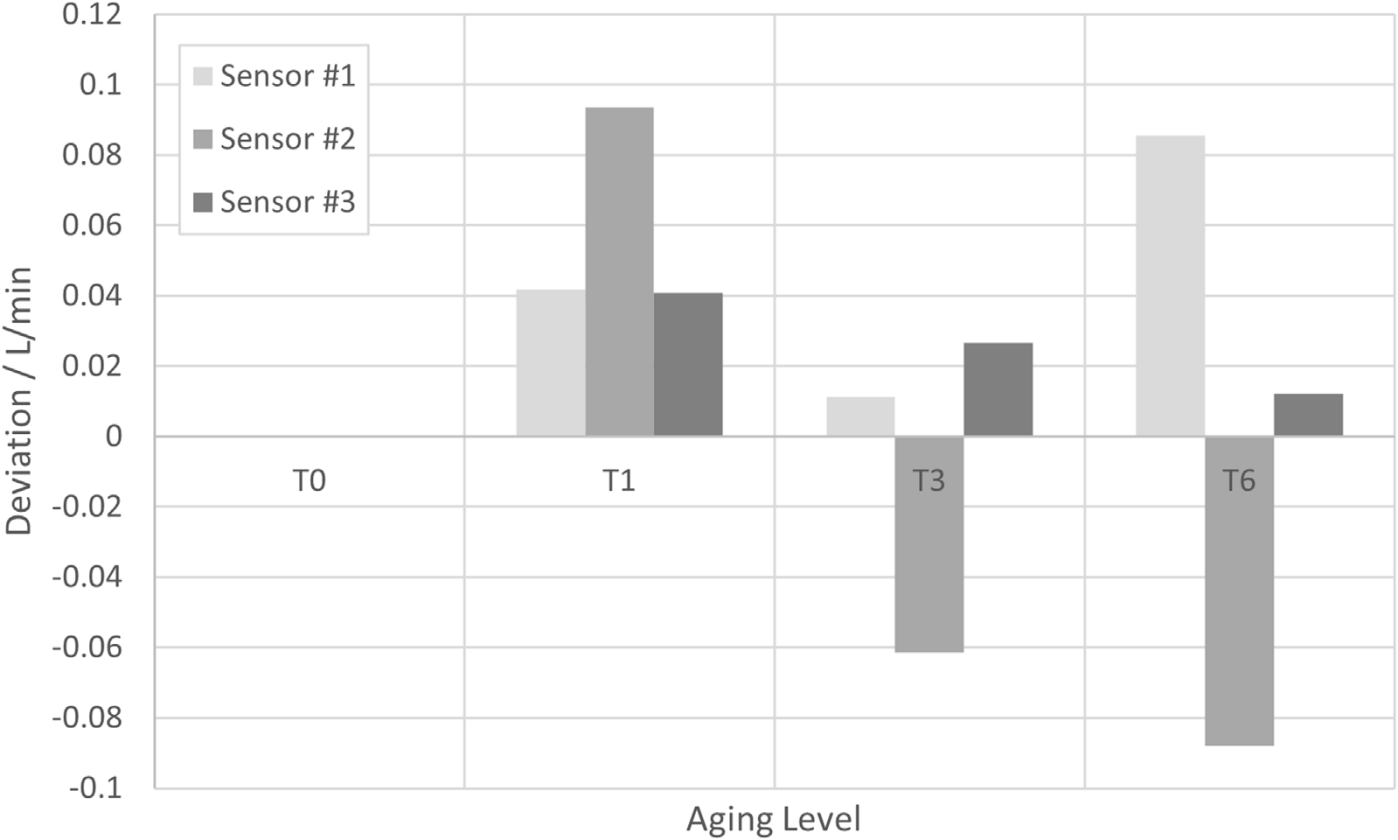
Deviations of the measurement result obtained at several aging levels with three different SU sensors from the result obtained with a reference sensor. In order to eliminate the systematic errors of the SU sensors, the respective deviation of the reference sensor at T0 has been subtracted.

Figure 8 shows the deviations of three SU sensors after the above-mentioned treatments to the results obtained with a reference sensor. For all measurements, the flow rate was again 10 L/min, the temperature 21.4°C (kept constant within ±0.2°C), and the pressure was 1.6 bar(g) [kept constant within ±0.4 bar(g)]. The deviation for T0 was subtracted from the other deviations in order to eliminate the systematic error of each SU sensor, with such subtracted deviations being ≤0.15 L/min for all three sensors.

Although whether there is a systematic error with increasing aging level cannot be clearly be analyzed from the data, a rather conservative estimation is made here: a Type B uncertainty with a half rectangular width of 0.1 L/min at a flow rate of 10 L/min and proportionality to the flow rate is assumed. Equation 5 yields a respective uncertainty contribution:

$$u_{t_{x}}\left(Q\right)=\frac{0.1\;L/\min}{\sqrt{3}}\cdot \frac{Q}{10\;\frac{L}{\min}}=\frac{0.01\cdot Q}{\sqrt{3}}.$$ (5)

### 5.3 Process parameter-related aspects

#### 5.3.1 Temperature

To investigate the uncertainty contribution of media temperature differences, measurements were carried out at different media temperatures within a chosen sensor calibration table (in this case, 22°C ± 5°C).

**FIGURE 9:**
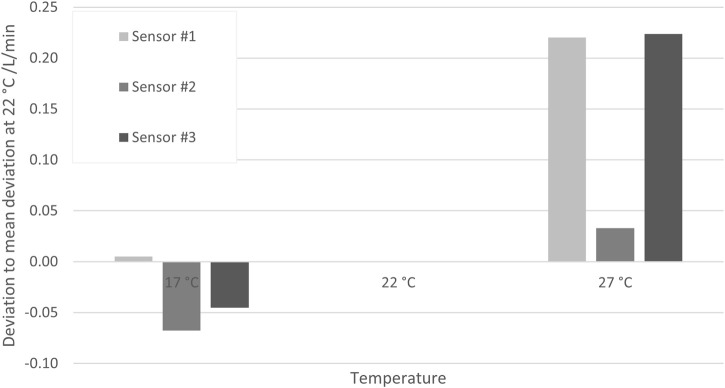
Deviations of the measurement result obtained at three temperatures with three different SU sensors from the result obtained with a reference sensor. In order to eliminate the systematic errors of the SU sensors, the respective deviation of the reference sensor at 22°C has been subtracted.

Figure 9 shows the deviations obtained with three SU sensors from the results obtained with the reference sensor at 17°C, 22°C, and 27°C (each kept constant within ±0.1°C during the respective measurements). To eliminate the systematic error of each SU sensor, the average deviation at 22°C was subtracted from the deviations, with such being ≤0.15 L/min in all cases. The pressure during all measurements was (1.6 ± 0.4) bar(g), and the set flow rate was 10 L/min.

Although the deviations are obviously larger at the higher temperature and smaller at the lower temperature, a conservative approach is again chosen by assuming that any deviation due to temperature is smaller than 0.25 L/min for all temperatures in the specified calibration range of 17°C–27°C. The BioPAT^®^ Flow sensor has three standard calibration tables, each for a specific temperature range. It is assumed that the results are analogous for the other calibration tables. Furthermore, temperature-dependent measurements at different flow rates (data not shown) indicate that the deviations are constant over all flow rates. Thus, Equation 6 shows that a Type B uncertainty contribution with a constant half-rectangular width of 0.25 L/min can be assumed as an uncertainty contribution:

$$u_{T}\left(Q\right)=\frac{0.25\;L/\min}{\sqrt{3}}=0.14\;\frac{L}{\min}.$$ (6)

#### 5.3.2 Different media

All previous measurements and considerations were made for water as the medium for which the flow rate is measured. With respect to the underlying measurement principle (time of flight of an ultrasound signal), it is, however, likely that for media other than water, other deviations and uncertainties would apply, as the sound velocity in such media and particularly its dependency on changes in temperature or pressure will be different. However, studies with water do not meet the requirements of all applications because the sensors may contain media with properties that differ from water (Bissig et al., 2023). While this generally would require a detailed investigation of the effects within a particular medium to consider, only one medium was used here instead to get an impression of the general effect of measuring a different medium than water. A 200 g/L BSA solution was prepared, and the flow rate measured with three BioPAT^®^ Flow sensors was compared to the results measured with a reference sensor. For all measurements, the flow rate was 10 L/min, the temperature was 21.6°C (kept constant within ±0.3°C), and the pressure was 1.5 bar(g) [kept constant within ±0.4 bar(g)].

**FIGURE 10:**
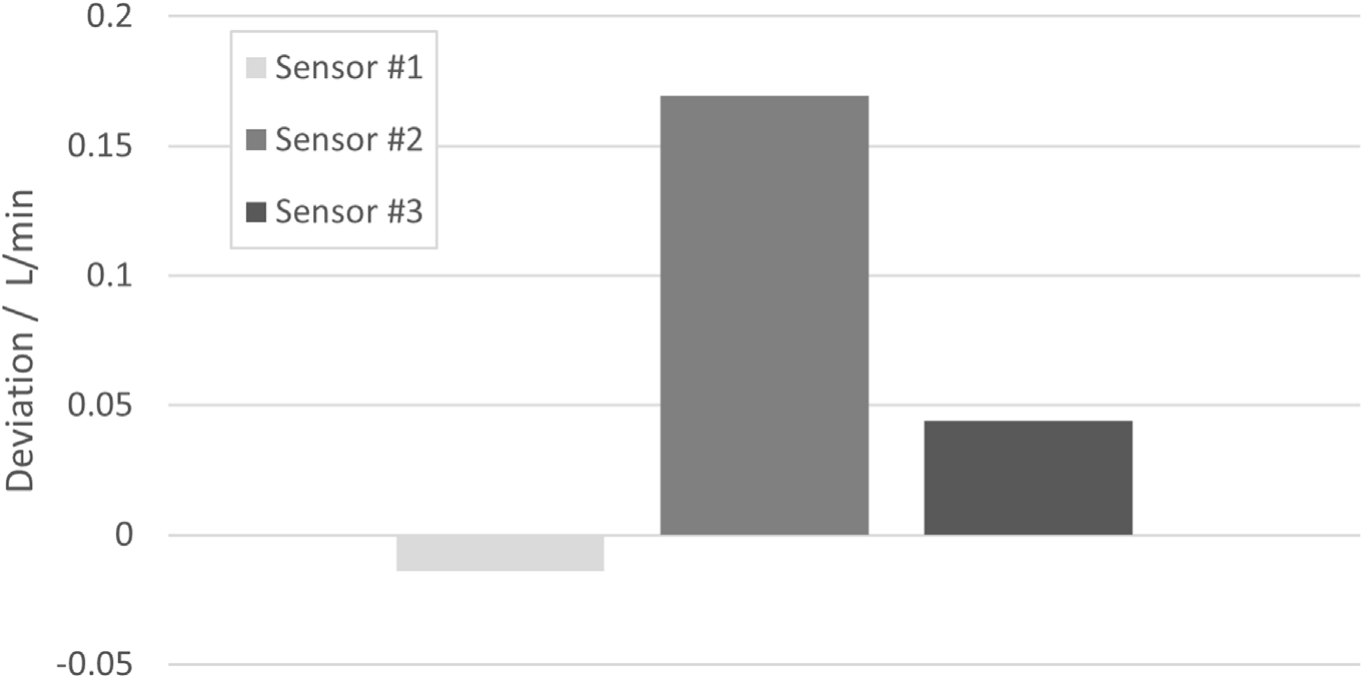
Deviations of the measurement result obtained in a 200 g/L BSA solution with three different SU sensors from the result obtained with a reference sensor. In order to eliminate the systematic errors of the SU sensors, the respective deviation of the reference sensor in water has been subtracted.

Figure 10 shows the deviations of three SU sensors to the results obtained with a reference sensor in the above-mentioned BSA solution. As in previous sections, the deviation for the respective measurement in water was subtracted from the other deviations in order to eliminate the systematic error of each SU sensor, with such subtracted deviations being ≤0.15 L/min for all three sensors.

As in the previous section, it cannot be clearly determined from these data whether there is systematic behavior. However, in view of the rather sparse data and the high relevance of fundamental medium properties for the measurement principle, a conservative approach is chosen in Equation 7 by assuming a Type B uncertainty with a half rectangular width of 0.2 L/min at a flow rate of 10 L/min and proportionality to the flow rate:

$$u_{M}\left(Q\right)=\frac{0.2\;L/\min}{\sqrt{3}}\cdot \frac{Q}{10\;\frac{L}{\min}}=\frac{0.02\cdot Q}{\sqrt{3}}.$$ (7)

#### 5.3.3 Volumetric flow rate

**FIGURE 11:**
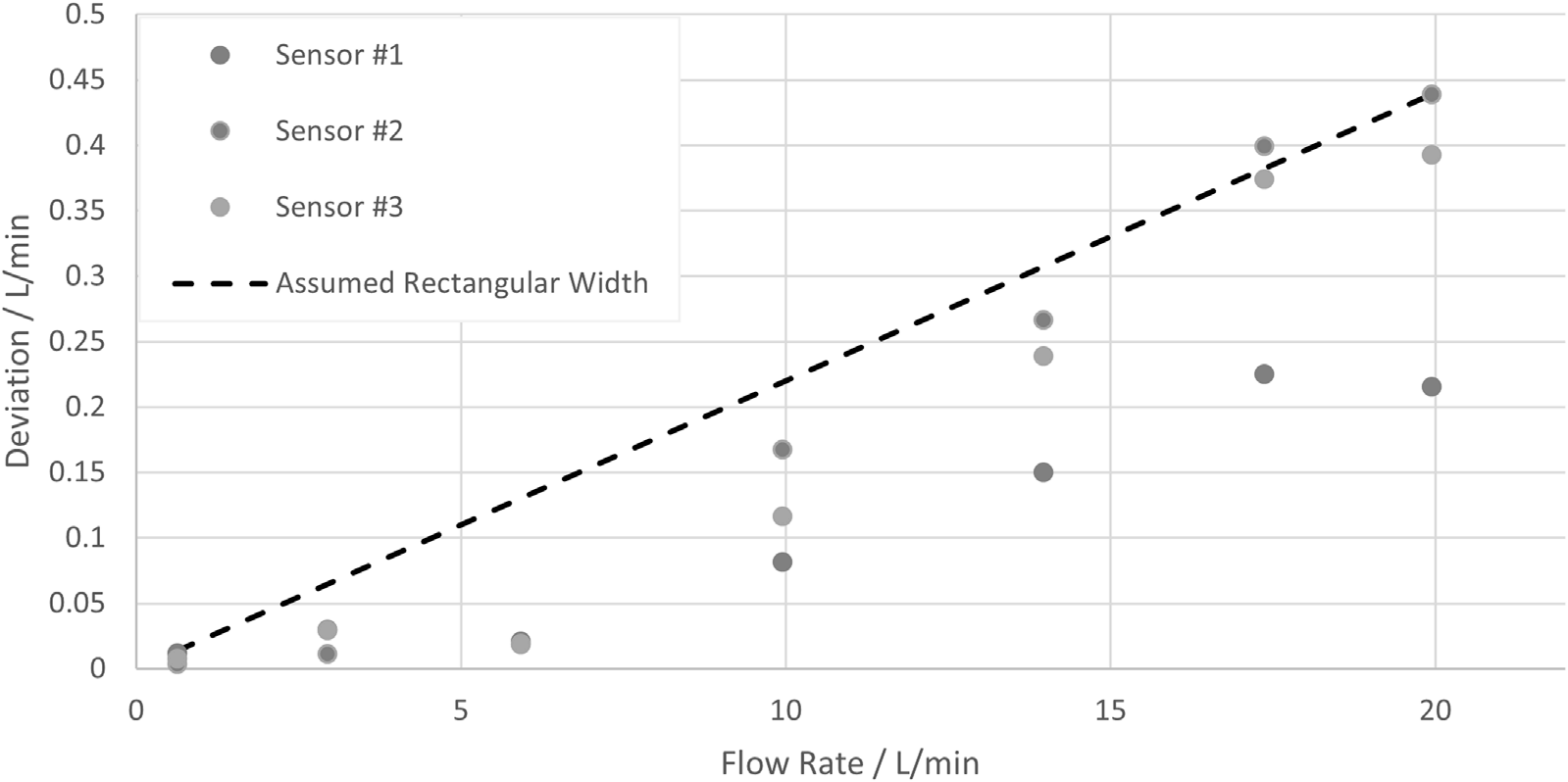
Deviations of the measurement result obtained at seven flow rates from the results obtained with a reference sensor and the assumed rectangular width.

The flow rate measured at the reference sensor is compared to the flow rate measured with three SU flow sensors, and the deviations are plotted in Figure 11. The temperature was 21.6°C (kept constant within ±0.4°C), the pressure was 1.7 bar(g) (kept constant within ±0.4 bar(g)), and seven set flow rates between 0.6 L/min and 19.9 L/min were considered.

The deviation increases with increasing flow rate. For simplification, the respective uncertainty contribution is assumed here to be proportional to the flow rate, and as a rather conservative value, a Type B uncertainty with a half rectangular width of 0.45 L/min at a flow rate of 20 L/min is assumed. Equation 8 yields the respective uncertainty contribution:

$$u_{Q}\left(Q\right)=\frac{0.45\;L/\min}{\sqrt{3}}\cdot \frac{Q}{20\;\frac{L}{\min}}=\frac{0.0225\cdot Q}{\sqrt{3}}.$$ (8)

#### 5.3.4 Pressure

Pressure levels between 0.6 bar(g) and 2.8 bar(g) were investigated. The temperature was 21.5°C (kept constant within ±0.2°C), and the set flow rate was 10 L/min.

**FIGURE 12:**
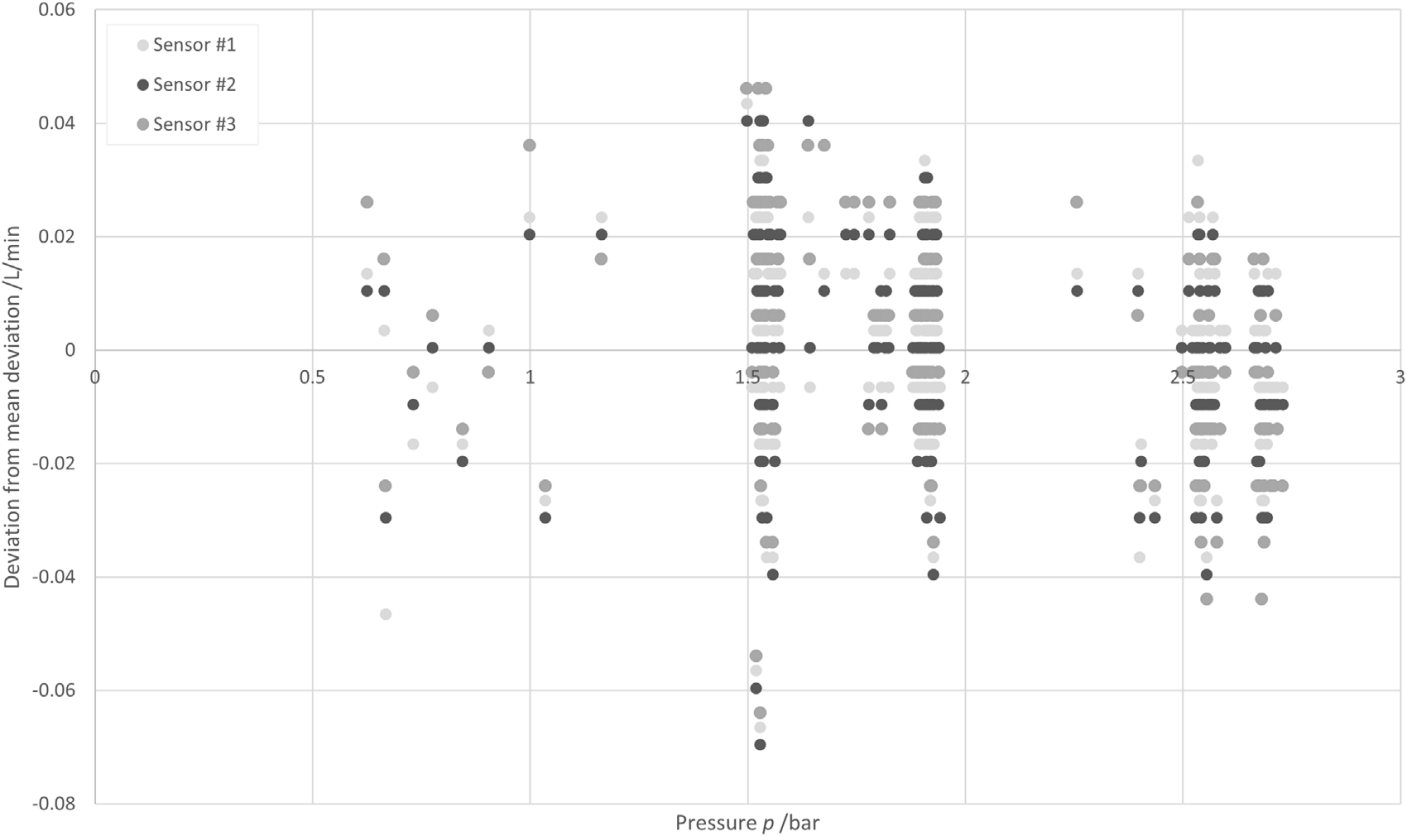
Deviations of the measurement results obtained at several pressures with three different SU sensors from the results obtained with a reference sensor.

Figure 12 shows the deviations obtained with the three SU sensors from the results obtained with the reference sensor at several pressures. Again, to eliminate the systematic error of each SU sensor, the average deviation was subtracted from the deviations, with such subtracted deviations being ≤0.15 L/min for all three sensors. No systematic relationship between the deviations and the pressure level was observable. Thus, pressure is not further considered in the measurement uncertainty analysis.

### 5.4 Combined standard uncertainty

The single uncertainty contributions were determined experimentally in this article. Inserting the results into Equation 4 yields Equation 9:

$$u\left(Q\right)=\sqrt{\begin{array}{c} {\left(\frac{0.01\cdot Q}{\sqrt{3}}\;\right)}^{2}+{\left(0.041\;\frac{L}{\min}\right)}^{2}+{\left(0.097\frac{L}{\min}\right)}^{2}\\ +{\left(0.033\frac{L}{\min}\right)}^{2}{+\left(u_{t_{x}}\left(Q\right)\right)}^{2}+{\left(u_{T}\left(Q\right)\right)}^{2}.\\ +{\left(u_{M}\left(Q\right)\right)}^{2}+{\left(u_{q}\left(Q\right)\right)}^{2}+{\left(u_{p}\left(Q\right)\right)}^{2} \end{array}}$$ (9)

**FIGURE 13:**
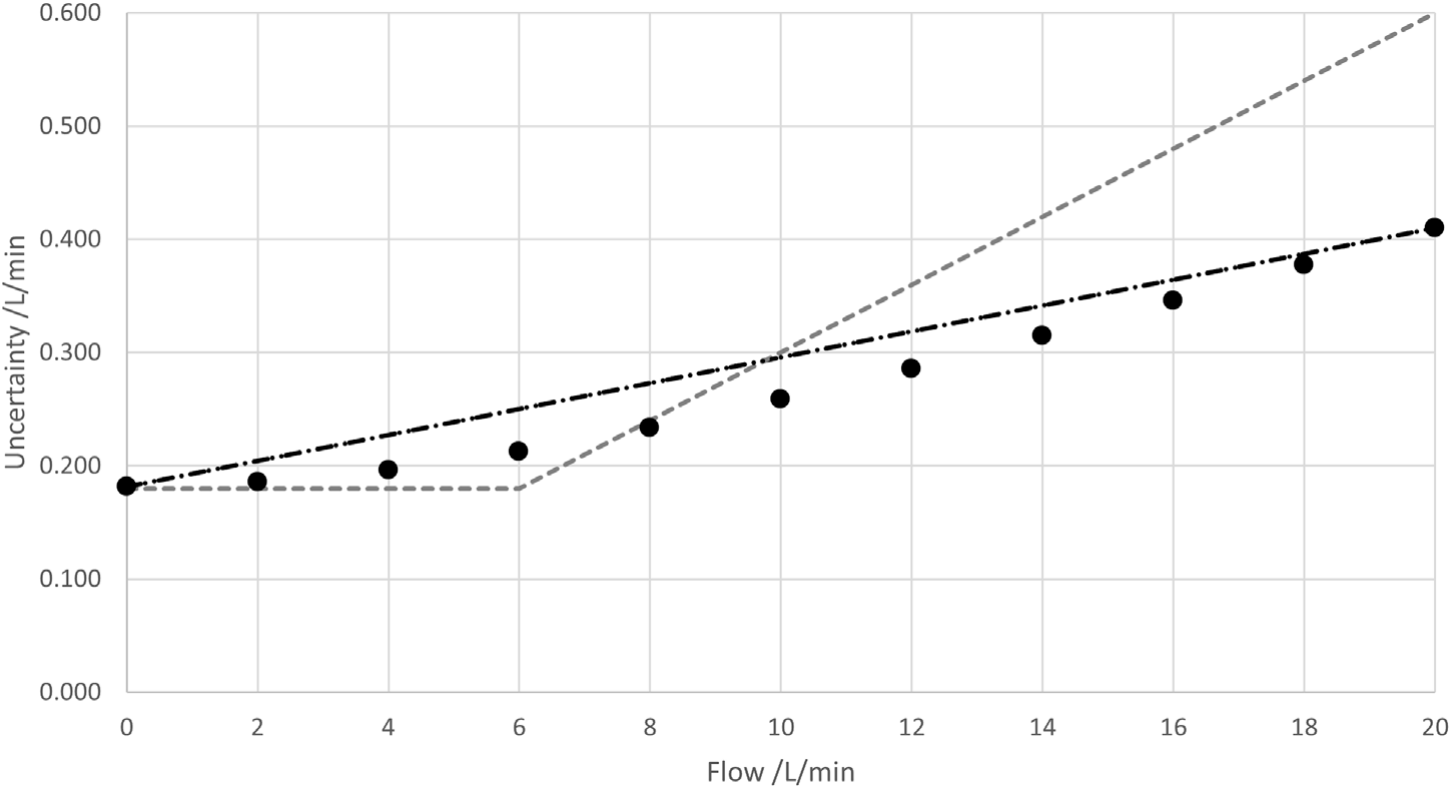
Combined standard uncertainty, calculated at 11 flow rates between 0 L/min and 20 L/min (black points), and a linear regression for the upper limit (dashed black line). Additionally, the sensor system accuracy stated in the datasheet of this sensor is shown (dashed gray line).

The combined standard uncertainty depends on the flow rate and was calculated at 11 flow rate levels: 0 L/min, 2 L/min, … , 20 L/min, yielding a nonlinear increase. Figure 13 shows the uncertainty calculated at these 11 points. A linear regression for an upper limit of the combined standard uncertainty was calculated as shown by the dashed black line, yielding Equation 10:

$$u\left(Q\right)=0.0114\;Q+0.182\;\frac{L}{\min}.$$ (10)

This corresponds to the more common expression of 0.91% of the full sensor range +1.14% of the measured value.

For comparison, the sensor system accuracy stated in the datasheet (see Table 2) is shown in Figure 13 as a dashed gray line.

**TABLE 2:** The sensor system accuracy, as stated in the datasheet of the SU sensor.

Sensor system accuracy	
3%–30% $Q_{\max}$	30%–100% $Q_{\max}$
0.18 L/min	3% c.v

The uncertainty determination from this work yields comparable results to the stated accuracy from the sensor datasheet. In contrast to this work, where the individual uncertainty contributions were separately investigated and finally combined, the accuracy statement in the sensor datasheet was derived from a statistical analysis where multiple impacts on the sensor uncertainty were investigated jointly. Multiple measurements at different temperatures, pressures, and flow rate levels were carried out with different combinations of flow pipes and clamp-on transmitters. Moreover, different process media and aging levels of SU sensor components were investigated. Thus, all impacting factors considered in this work were included.

For each measurement, the deviation of the sensor value to a reference sensor was calculated using Equation 11:

$$∆Q=Q_{\text{Flow}-\text{Pipe}}-Q_{\text{Reference}.}$$ (11)

To derive the sensor accuracy, the mean deviation from these multitudes of measurements to the reference sensor and the standard deviation of the deviations to the reference values were calculated. Both values were added to calculate the 1-sigma accuracy as shown in Equation 12:

$$“1‐\text{sigma accuracy}”=\left|\text{mean of}∆Q\right|+\text{standard deviation of}∆Q.$$ (12)

The stated sensor accuracy was now chosen in a way that the calculated 1-sigma accuracy was lower than the stated sensor accuracy under all investigated process conditions.

The combined uncertainty, as stated above, means that with a coverage interval (also known as the confidence interval) of 68.27%, the true value is within the range of ± u(Q) around the measured value, assuming that the sensor deviations are distributed normally.

### 5.5 Combined expanded uncertainty

It is good practice in metrology to state expanded uncertainties with a coverage interval of 95.45%. For that purpose, the combined standard uncertainty is multiplied with an expansion factor *k* that can be assumed here as *k* = 2 with respect to the high number of contributions considered and no dominant Type B contributions. One of the advantages of the dedicated uncertainty determination presented in this work is that the “level of security” can be adjusted via the coverage factor/interval. For example, an expansion factor of *k* = 3 can be employed to obtain a coverage interval of 99.73%.

**FIGURE 14:**
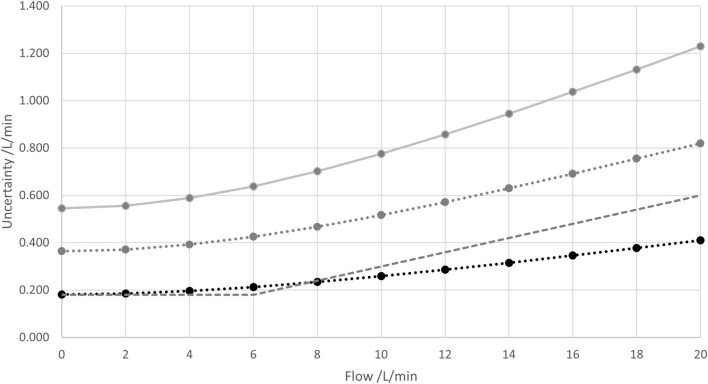
Combined standard uncertainty and (absolute of the) “1-sigma accuracy” (same symbols/colors as in Figure 13) together with combined expanded uncertainties U95.45% obtained with an expansion factor of k = 2 (gray) and U99.73% obtained with an expansion factor of k = 3 (bright gray). Lines are drawn to guide the eyes.

The results for the calculated expanded uncertainties with the different expansion factors are shown in Figure 14 below.

## 6 Discussion and outlook

A dedicated determination of the measurement uncertainty for an SU flow sensor was presented in this work. The calibration uncertainties of both the reference sensor and the calibration of the SU sensor with this reference sensor were considered, as well as process parameter-related aspects (pressure, temperature, and flow rate). A particular focus was laid on the SU characteristics by investigating the reproducibility of the sensor, the flow pipe, the coupling process, and the component age. Finally, the influence of the medium was studied, though to a limited extent.

To get an impression of the individual contributions to the measurement uncertainty, an exemplary pie chart is shown in Table 3. It indicates the uncertainty contributions calculated at 2 L/min, 10 L/min, and 20 L/min flow rates. At lower flow rates, factors that were assessed to be not flow rate dependent (reproducibility terms) have larger proportions than the other factors, while the opposite is the case for larger flow rates.

**TABLE 3:** Uncertainty contributions at 2 L/min, 10 L/min, and 20 L/min.

Flow rate/uncertainty contribution	2 L/min	10 L/min	20 L/min
Ref. sensor calibration	<0.1%	<0.1%	<0.1%
Vol. flow rate	2%	25%	40%
Repr. coupling	3%	2%	1%
Repr. transmitter	27%	14%	6%
Repr. SU comp	5%	3%	1%
Instrumental drift	<0.1%	5%	8%
Diff. media	2%	20%	32%
Temperature	61%	31%	12%

An advantage of the presented approach to determining the sensor uncertainty is that much more information about the contributing factors is generated, and it is clearer which factors impact the uncertainty most. This allows specialized sensor applications and gives valuable indications about how the sensors could be optimized to achieve a better accuracy performance.

For example, it can be noted that the uncertainty of the sensor system cannot be improved by using a better reference sensor. Moreover, it can be observed that at 10 L/min, the SU sensor-related uncertainty contributions (reproducibility terms and instrumental drift) account for approximately a quarter of the total sensor uncertainty.

For the example shown, temperature variations contribute most to the sensor uncertainty. If processes could, for example, be run at constant temperatures, the resulting sensor uncertainty could be roughly cut by 1/3 if a specific calibration were used. Similar considerations can be made for the other factors impacting the sensor uncertainty.

The presented approach could be used as a foundation to generate sensor uncertainty information that is comparable between different sensor manufacturers. As no consistent ruleset for determining the uncertainty of SU sensors currently exists, each manufacturer decides on its own how to assess and present this information, which is confusing for the users of the SU sensors. Thus, we recommend evaluating whether this approach can be transferred to investigate the measurement uncertainty of other SU sensors. For digitalization efforts involving single-use sensors to succeed, establishing standardized industry guidelines for sensor characterization can play a crucial role. Digitalization depends not only on advanced algorithms but also on the accessibility of dependable data. Thus, we recommend evaluating whether this approach can be transferred to the investigation of the measurement uncertainty of other SU sensors.

## Data Availability

The raw data supporting the conclusions of this article will be made available by the authors, without undue reservation.
